# GC-MS Analysis of the Composition of the Extracts and Essential Oil from *Myristica fragrans* Seeds Using Magnesium Aluminometasilicate as Excipient

**DOI:** 10.3390/molecules24061062

**Published:** 2019-03-18

**Authors:** Inga Matulyte, Mindaugas Marksa, Liudas Ivanauskas, Zenona Kalvėnienė, Robertas Lazauskas, Jurga Bernatoniene

**Affiliations:** 1 Department of Drug Technology and Social Pharmacy, Medical Academy, Lithuanian University of Health Sciences, Kaunas LT-50161, Lithuania; inga.matulyte@lsmu.lt (I.M.); zenona.kalveniene@lsmuni.lt (Z.K.); 2 Department of Analytical and Toxicological Chemistry, Medical Academy, Lithuanian University of Health Sciences, Kaunas LT-50161, Lithuania; mindaugas.m.lsmu@gmail.com (M.M.); Liudas.ivanauskas@lsmuni.lt (L.I.); 3 Institute of Pharmaceutical Technologies, Medical Academy, Lithuanian University of Health Sciences, Kaunas LT-50161, Lithuania; 4 Institute of Physiology and Pharmacology, Medical Academy, Lithuanian University of Health Sciences, Kaunas LT-50161, Lithuania; Robertas.lazauskas@lsmuni.lt

**Keywords:** *Myristica fragrans*, nutmeg, essential oil, extract, magnesium aluminometasilicate, hydrodistillation

## Abstract

*Myristica fragrans* (f. Myristicaceae) seeds are better known as a spice, but their chemical compounds may have a pharmacological effect. The yield of their composition of extracts and essential oils differs due to different methodologies. The aim of this study was to evaluate an excipient material—magnesium aluminometasilicate—and to determine its influence on the qualitative composition of nutmeg extracts and essential oils. Furthermore, we wanted to compare the yield of essential oil. The extracts were prepared by maceration (M) and ultrasound bath-assisted extraction (UAE), and the essential oil—by hydrodistillation (HD). Conventional methods (UAE, HD) were modified with magnesium aluminometasilicate. The samples were analyzed by gas chromatography-mass spectrometry (GC-MS) method. From 16 to 19 chemical compounds were obtained using UAE with magnesium aluminometasilicate, while only 8 to 13 compounds were obtained using UAE without an excipient. Using our conditions and plant material, for the first time eight new chemical compounds in nutmeg essential oil were identified. Two of these compounds (γ-amorphene and cis-α-bergamotene) were obtained with the use of excipient, the other six (β-copaene, bergamotene, citronellyl decanoate, cubebol, cubenene, orthodene) by conventional hydrodistillation. Magnesium aluminometasilicate significantly increased the quantity of sabinene (from 6.53% to 61.42%) and limonene (from 0% to 5.62%) in essential oil. The yield of the essential oil from nutmeg seeds was significantly higher using magnesium aluminometasilicate; it increased from 5.25 ± 0.04% to 10.43 ± 0.09%.

## 1. Introduction

Since ancient times, nutmeg (*Myristica fragrans*, f. Myristicaceae) has been used as a spice. Its aphrodisiac effect is often mentioned [1]. In traditional medicine, nutmeg is used to treat rheumatism, pain, nausea, stomach cramps, and other illnesses [2]. A lot of research is being done in order to test the pharmacological effects of nutmeg. Scientific sources say that nutmeg has antibacterial, insecticidal, antioxidant, anticancer, antidepressant, hepatoprotective, and many other effects [3,4,5,6]. The most common studies about essential oils are of nutmeg, which is rich in terpenes, phenols, various organic acids, and other compounds. Using different solvents, methods, and conditions, the composition of extracts and essential oils variations were studied [3,7,8,9]. Nutmeg essential oil is colorless to pale-yellow with a specific odor. The essential oil yield may be more than 10% [10] and varies by 5–15% [11]. *Myristica fragrans* essential oil has predominantly monoterpene hydrocarbons (α-thujene, β-pinene, sabinene), sesquiterpene hydrocarbons (d-germacrene, trans-β-bergamotene), monoterpene alcohols (linalool, α-terpineol, *cis*-p-menth-2-en-1-ol, terpinen-4-ol), esters (α-terpinyl acetate, *cis*-sabinene hydrate acetate, citronellyl acetate), aromatics (eugenol, myristicin, elemicin, methoxyeugenol), and other compounds [5,7,8,9,12]. 

Traditional methods such as maceration, percolation, infusion, decoction, and Soxhlet method are commonly used to produce extracts [8,13]. These methods are cheap but less efficient, which wastes a lot of time and raw materials [14]. One of the more recent methods for extracts is extraction in an ultrasound-assisted bath, it is an effective method, in which it is possible to choose the desired conditions (i.e., temperature, duration), and it saves on materials and time [2,15]. Ultrasound-assisted extraction (UAE) has many modifications with solvents, and extra material can be used for extraction; for example: surfactants [16], polysaccharides [17], salts [18], acids [19], and other excipients, which can increase the efficiency of extraction.

The most popular methods for extracting essential oils are distillation with water (hydrodistillation) and distillation with steam or steam and water [12,14,20]. Other methods are cold or hot-pressing, supercritical fluid extraction, enfleurage, microwave, and ultrasound-assisted methods, solvent extraction, and aqueous infusion [18,21].

Hydrodistillation is a method used for extracting essential oils. It is cheap because it mostly uses water as a solvent. The equipment is not expensive, most commonly a Clevenger-type apparatus or similar modifications are used [14,20]. In different sources, hydrodistillation is carried out for various times and solid:solvent ratios. Hydrodistillation is carried out using water, and few studies have been carried out using extra material for extraction such as sea water [22], non-ionic surfactants [23] or 5% NaCl salt solution. [14]. According to the information found, it can be said that excipient substances are rarely used for hydrodistillation. 

Excipients are used for improving extraction and changing environmental conditions. For example, salts that change ion voltage, surfactants, emulsifiers (sorbitan esters (Spans^®^), polysorbates (Tweens^®^)), and pH-adjusting substances are used [9,24,25]. The magnesium aluminometasilicate will be used for the first time as an excipient for chemical compound extractions, previously it was used as an excipient only in solid dosage forms [26,27]. It is a white amorphous powder with high surface area, practically insoluble in water. The magnesium aluminometasilicate has the ability to absorb materials, which is characterized by water absorption capacity [26,28].

The purpose of this study is to evaluate excipient material—magnesium aluminometasilicate— and to determine its influence on the qualitative composition of nutmeg extracts and essential oils. Furthermore, to compare the yield of essential oil using conventional hydrodistillation and hydrodistillation with magnesium aluminometasilicate.

## 2. Results

Using the traditional maceration method (conditions are mentioned in Table 1, see Materials and Methods) myrislignan and elemicin were established at 22.59% and 13.99%, respectively, (Table 2). Maceration as a method was modified with magnesium aluminometasilicate (0.5%, 1%, and 2% excipient), but the results were not significant. The magnesium aluminometasilicate did not increase the quantity of chemical compounds, so these conditions and results were not mentioned in the research.

Using other extraction techniques (i.e., UAE, HD), myrislignan and elemicin were not found. The extract (M1) has 10 different active compounds: *α*-phellandrene, *β*-myrcene, *γ*-asarone, elemicin, and others (Table 2).

Analyzing the influence of ethanol concentrations on the extraction of compounds, it was found that most of the different compounds were extracted using 70% ethanol (fifteen compounds), or at least 50% ethanol (eight compounds). The higher the ethanol concentration, the better the extracts were extracted, for example with *α*-pinene, 2.4%, 8.3%, and 14.67%, respectively (Table 2). In contrast, when weaker ethanol concentrations were used more isolemicin was extracted (62.24%, 23.63%). In the extract of 96% ethanol isolemicin was not found.

The data of the extracts with magnesium aluminometasilicate, using 70% ethanol as a solvent (UAE4, UAE5, UAE6, Table 2) showed that when 1% magnesium aluminometasilicate was used, the utmost quantity of compounds was identified (90.92%). Magnesium aluminometasilicate helped extract sabinene (from 29.9 to 32.69%, in extracts without magnesium aluminometasilicate sabinene was not found). The following compounds were also detected in extracts with magnesium aluminometasilicate exposure: myrcene, isoterpinolene, isogermacrene, limonene, *cis*-sabinene hydrate, *γ*-terpinene, *γ*-amorphene, and *β*-myrcene (Table 2).

**Figure 1:**
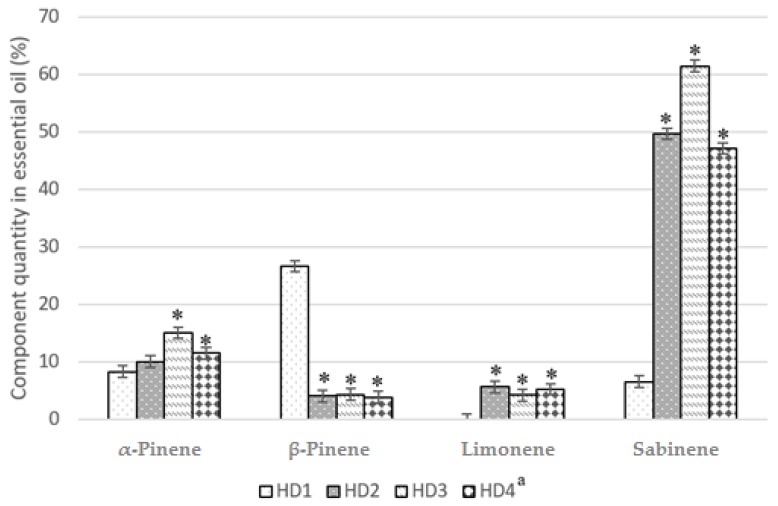
Magnesium aluminometasilicate’s influence on the amount of *α* and *β*-pinene, limonene, and sabinene in the essential oils of nutmeg. Component quantity in essential oils, * *p* < 0.05 versus hydrodistillation without magnesium aluminometasilicate. ^a^ Sample code can be seen in Table 1.

The composition of the essential oils, which were obtained using hydrodistillation (HD1) and hydrodistillation with excipients from HD2 to HD4 (Table 1), showed that 1% of magnesium aluminometasilicate significantly improved amount of *α*-pinene and sabinene compared to conventional hydrodistillation, respectively 54.95% and 106.32%. Using 0.5% and 2% of magnesium aluminometasilicate the amount of the *α*-pinene and sabinene was higher than HD1 but lower than HD3 (Figure 1). 

In HD1, essential oil consisted of β-copaene, bergamotene, bicyclogermacrene, cubebol, cubenene, and piperitol, and these compounds were not identified in other extracts. Ten compounds using magnesium aluminometasilicate were obtained, and in comparison to the essential oil of HD1, their amount was not large but it was about 13.05 ± 3.54% of the quantity of all of the identified compounds in essential oil.

Comparing water and ethanol as solvents, and maceration with ultrasound-assisted extraction methods, it was found that water is better in extracting *α*-phellandrene (13.04%) than 70% ethanol (1.41%), but worse in γ-asarone (0.79% versus 2.07%), *cis*-p-menth-2-en-1-ol (0.43% versus 5.88%), 4-carene, trans-(+)-(3.37% versus 6.56%) (Table 2).

**Figure 2:**
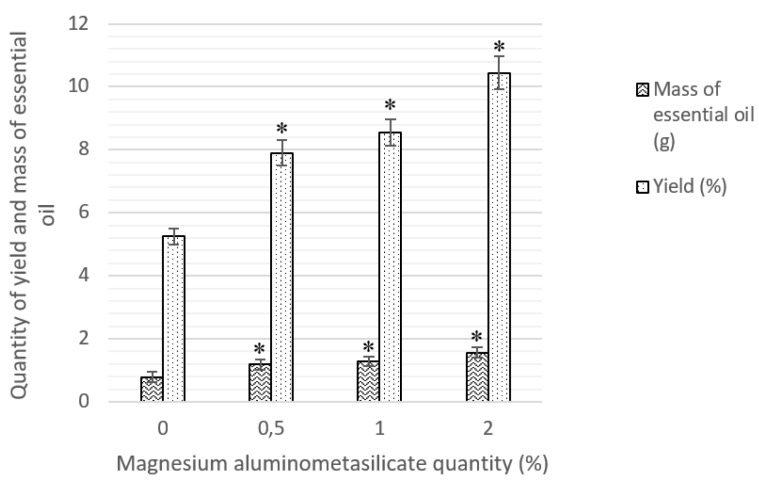
Essential oils’ mass and yield from nutmeg using HD and HD with magnesium aluminometasilicate. Mass and yield of essential oil, * *p* < 0.05 versus hydrodistillation without magnesium aluminometasilicate.

By using hydrodistillation from nutmeg (HD1), 0.79 ± 0.04 g of essential oil was obtained (Figure 2).

At least this amount of mass was gained in all of the samples. Then we used the magnesium aluminometasilicate, and the essential oil was obtained in higher quantities—from 1.19 ± 0.09 g to 1.57 ± 0.09 g (*p* < 0.05). The highest yield of essential oil (HD4, 10.43%) was obtained using 2% magnesium aluminometasilicate (Figure 2).

## 3. Discussion

In this study, the extracts, essential oil, and the influence of excipient were analyzed. The composition of the aqueous (M1) and ethanolic (UAE1–UAE6) extracts and essential oil (HD1–HD4) of nutmeg were determined (Table 1). Our results show that the extract which was obtained using maceration with water had 10 different chemical compounds of which the most commonly found are myrislignan (22.59%), elemicin (13.99%), and α-phellandrene (13.04%). We did not find any research done with aqueous extract of nutmeg. In ethanolic extract, which was made by using maceration, the quantity of elemicin (5.7%) was 2.45 times lower, and the quantity of α-phellandrene was 21.73 times higher [8]. Elemicin has antibacterial effects [29] and α-phellandrene, anti-inflammatory [30], so using maceration with water is more effective if a bigger effect is needed. Myrislignan has anti-inflammatory [31] and anti-cancer [32] effects. This compound was not extracted using ethanol [8].

In our study, when 96% ethanol was used (ultrasound extraction, sample UAE3), the α-pinene content was obtained at 14.67%, in other study using the same method, 6.75 ± 0.92% was obtained [8]. In one of the studies, the compound of *cis*-p-menth-2-en-1-ol was obtained at about 0.1% [8]. However, in our study using 96% ethanol the compound was obtained at 3.25%, which is 32.5 times more than in the previously mentioned study, and if we compare this with extraction using 70% ethanol, the yield of *cis*-p-menth-2-en-1-ol was obtained at 58.8 (5.88%) times more in our research (Table 2). Using nutmeg seeds material from Grenada we did not find any isoelemicin (ultrasound extraction, 96% ethanol), the other researchers found 0.8 ± 0.46% [8], but we used 50% and 70% ethanol and determined the quantity of isoelemicin, which was 62.24% and 23.63%, respectively. The excipients are used to improve the extraction of the active compounds. Surfactants used in experiments improve the extraction of certain compounds, such as Sodium dodecyle sulfate (SDS), Triton X 100, PEG 2000, Brij 35, Tween, etc. [25]. In our study we used magnesium aluminometasilicate as excipient, and our purpose was to research and determine the effect of magnesium aluminometasilicate on the extraction of active compounds. Previously, this substance has not been used in studies with extracts and essential oils. Magnesium aluminometasilicate is used as excipient in solid drug form formulations [26,27,33]. Using this material, we have relied on its mechanism of action. It adsorbs high loads of oil [34], so we predicted that it could extract more essential oil components. The results of GC-MS analysis showed the influence of the excipients on gaining different quantities of certain compounds. Magnesium aluminometasilicate significantly improved the amount of sabinene. If we compare it to the essential oils that were made by using simple hydrodistillation (Table 2), we see that 0.5% and 2% of magnesium aluminometasilicate increased sabinene by 7.41 ± 0.2 times (49.6% and 47.1%, traditional hydrodistillation—6.53%), the 1% of magnesium aluminometasilicate increased sabinene by 9.41 times (61.42%). These results are significant. Dupuy [7] reported 6.00–36.08% quantity of sabinene, and the nutmeg seeds were from Indonesia. Also, magnesium aluminometasilicate had influence on limonene (Figure 1) and *γ*-terpinene extraction. The other samples made without the excipient did not have these compounds (M1, HD1, UAE1-UAE3) (Table 2). Sabinene has anti-inflammatory, antifungal, and antioxidant effects [35,36], limonene has low-toxicity and anticancer effect [37], and *γ*-terpinene characterizes as an antimicrobial and antioxidant agent [38]. Magnesium aluminometasilicate’s influence on *α*-pinene and *β*-pinene was different. The 1% of magnesium aluminometasilicate increased the quantity of *α*-pinene by 45% (from 8.27% to 15.05%) and decreased *β*-pinene by 84% (from 26.61% to 4.32%). Piaru [9] results showed that *Myristica fragrans* seeds’s essential oil from Malaysia contained 8.5% *α*-pinene and 3.5% *β*-pinene. 

**Figure 3:**
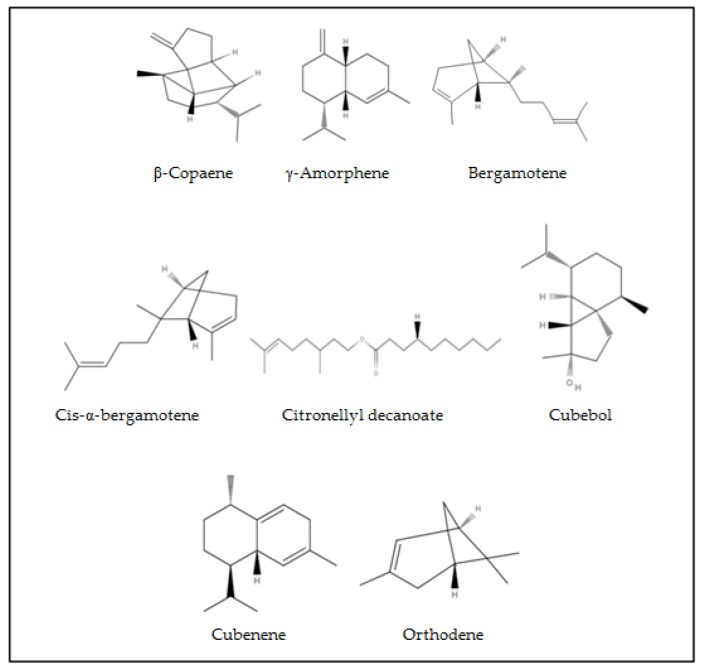
First time extracted and identified chemical compounds in essential oil of nutmeg.

After analyzing many studies with nutmeg essential oil we found a few compounds which were not mentioned in publications. Our study showed that essential oil HD1 from nutmeg (Grenada) contains *β*-copaene (0.25%), bergamotene (0.07%), citronellyl decanoate (0.3%), cubebol (0.05%), cubenene (0.07%), and orthodene (0.51%) (Figure 3).

In other research we found that the oleoresins of *Myristica fragrans* (Egypt) have α-copaene (0.63%) [8] and trans-*α*-bergamotene (0.1%) [39]. The compound of cubebol was found in *Piper Cuberica Linn*. essential oil and oleoresins [40] and *Chromolaena odorata* essential oil (8.6%) [41]. Cubenene was obtained in *Caryopteris incana* essential oil (9.7%) [42]. The *γ*-amorphene (0.03–0.78%) and *cis*-*α*-bergamotene (0.08–0.1%) were obtained by using magnesium aluminometasilicate in hydrodistillation (HD2–HD3) (Figure 3).

Volatile compound composition in extracts which have been produced using magnesium aluminometasilicate have similarities with essential oil. Seventy-one percent of the chemical compounds in the extract correspond to the composition of essential oil. However, only extracts with magnesium aluminometasilicate extracted β-myrcene and isoelemicin, and these compounds are not present in essential oil.

Our experimental data showed that the essential oil yield increased if we used magnesium aluminometasilicate as excipient which facilitated volatile compounds extraction. Essential oil yield from *Myristica fragrans* was 5.25 ± 0.04%; however, the magnesium aluminometasilicate was used for the yield of the essential oil and it was from 7.9 ± 0.09% to 10.43 ± 0.09%. Using sea water, the yield of oil-bearing rose increased statistically insignificantly by 0.045%, but the sea water significantly decreases the citronellol rate from 41.49% to 33.56%, and significantly increases geraniol (from 17.58% to 27.44%) [22]. Using non-ionic surfactants for extraction of sage essential oil it was found that Tween 20 increased the yield of oil at 1.83% and Span 80 or Span 20 had no effect [23]. Using 5% NaCl salt solution for hydrodistillation, the yield of lavender oil was 5.01 ± 0.45% in comparison with only water, where the yield was 4.55 ± 0.14% [14]. All essential oil samples with magnesium aluminometasilicate have 21 chemical compounds, although essential oil made by simple hydrodistillation has 24 compounds but that is not significantly important. The yield of nutmeg essential oil significantly increases with magnesium aluminometasilicate as excipient. The essential oil yield from nutmeg seeds (Nigeria) was 1.46% [43], from other nutmeg seeds (China) it was 2.4% [44]. Nutmeg seeds from Indonesia were hydrodistillated and the yield of essential oil was obtained at 5.84%.

## 4. Materials and Methods 

### 4.1. Materials

The dried seeds of *Myristica fragrans* (nutmeg) were from Grenada. The seeds were identified by Jurga Bernatoniene, Medical Academy, Lithuania University of Health Sciences, Kaunas, Lithuania. A voucher specimen (I 18922) was placed for storage at the Herbarium of the Department of Drug Technology and Social Pharmacy, Lithuanian University of Health Sciences, Lithuania. The seeds were a brown-beige color, had a characteristic odor, and strong, bitter, and spicy flavor. Dried nutmeg seeds were ground into a powder (particles smaller than 0.5 mm) with a laboratory mill. The samples were kept in an airtight container in a dark environment and in ambient temperature.

Magnesium aluminometasilicate (Neusilin^®^ US2, Fuji Chemical Industries Co., Ltd., Toyoma, Japan) was used as excipient. Ethanol 96% (Vilniaus degtine, Lithuania) was used as solvent for extraction. Distilled water was used throughout the experiment.

### 4.2. Maceration

The nutmeg seeds’ powder was extracted using a maceration technique with water in ambient temperature while occasionally stirring. The process lasted 3 days. Powder and solvent ratio were 1:20 (w/v). The extracts were filtered with paper filter (0.22 µm pore size) and stored in refrigerator at 4 °C until further analysis. Conditions are mentioned in Table 1.

### 4.3. Ultrasound Assisted Extraction 

Extracts were also prepared by using ultrasound-assisted extraction. This method was used as a simple and effective method for nutmeg extracts. The 1 g material and 20 mL solvent were used. We used different concentrations of ethanol for extraction (96%, 70%, and 50%). The flask with substance was put in an ultrasound bath (Sonorex Digitec DT 156 BH, Germany). Extracts were prepared on 25 °C temperature for 30 min. After that, the extracts were filtered with paper filter and stored in a refrigerator at 4 °C until further analysis.

Some samples were modified and made with magnesium aluminometasilicate. The extracts were made in the same conditions which were listed earlier. But for the solvent only a certain concentration of ethanol (70%) was used and the excipient was added in the mixture. The excipients’ concentration was from 0.5% to 2%. Magnesium aluminometasilicate (g) was based on ethanol quantity. Extracts were filtered and stored. Conditions are mentioned in Table 1.

### 4.4. Hydrodistillation 

Samples were prepared from nutmeg seeds and purified with water. The solid material was 15 g and the water was 300 mL. A Clevenger-type apparatus (European pharmacopoeia) for hydrodistillation was used. Hydrodistillation was carried out for 4 h. A colorless essential oil with specific odor was obtained. The essential oil was collected with water in airtight bottle and stored in a refrigerator at 4 °C until needed.

The hydrodistillation method was modified and magnesium aluminometasilicate as excipient was used. The amount of magnesium aluminometasilicate was based on water quantity, its concentration was from 0.5% to 2%. Fifteen grams of nutmeg seed powder were used and all of the sample ratios of solvent were the same (20:1). The essential oil was obtained and stored in the same conditions. Conditions are mentioned in Table 1.

### 4.5. Gas Chromatography-Mass Spectrometry Analysis

Gas chromatography-mass spectrometry analysis was performed on a GCMS-QP2010 system (Shimadzu, Tokyo, Japan). Twenty microliters of sample (extract or essential oil) was diluted to 1 mL with hexane (≥99%, Sigma–Aldrich, Germany). The column used was a 30 m × 0.25 mm i.d. × 0.25 µL film thickness RTX-5MS column. Flow rate of helium (99.999%, AGA Lithuania) carrier gas was set at 1.23 mL/min. The oven temperature was maintained at 40 °C for 2 min after injection and then programmed at 3 °C/min to 210 °C, at which the column was maintained for 10 min. The split ratio was 1:10. The mass detector electron ionization was 70 eV. Identification of volatile compounds was carried out using mass spectra library search (NIST 14) and compared with the mass spectral data from literature [45].

### 4.6. Statistical Analysis

Data are presented as mean ± SEM. Statistical analysis was preformed using Student’s *t*-test. The results were significant when *p* < 0.05.

## 5. Conclusions

Extracts and essential oils from *Myristica fragrans* seeds (Grenada) were prepared using three different methods (M, UAE, HD). The volatile compounds were analyzed by GC-MS. The results showed the samples’ quality and quantity of the chemical compounds composition. During the experiment magnesium aluminometasilicate was used for extraction in the hydrodistillation process. Previously, this excipient was used for the production of solid dosage forms. Ten chemical compounds were obtained using maceration, from eight to thirteen compounds were obtained using ultrasound-assisted extraction with different ethanol concentrations and without excipient, using UAE with magnesium aluminometasilicate, 16–19 compounds were obtained. Hydrodistillation with magnesium aluminometasilicate lead to 21 compounds, and finally, 24 compounds were obtained without the use of excipient. Although using hydrodistillation with the excipient fewer chemical compounds were obtained, some of the compounds’ quantities were bigger than using hydrodistillation without excipient. Magnesium aluminometasilicate as excipient improved the extraction of *α*-pinene, limonene, isoterpinolene, and sabinene, although it reduced the quantity of *β*-pinene. For the first time, eight chemical compounds in nutmeg essential oil were identified. Two of these compounds (*γ*-amorphene and cis-*α*-bergamotene) were obtained by the use of magnesium aluminometasilicate.

The yield of the essential oil from nutmeg seeds was significantly higher using magnesium aluminometasilicate. The data shows that essential oil yield which was obtained by hydrodistillation with water was 5.25 ± 0.04% and it increased using hydrodistillation with water and 2% of magnesium aluminometasilicate to 10.43 ± 0.09%. Using the 2% of magnesium aluminometasilicate the yield of essential oil increased by almost twice.

The use of magnesium aluminometasilicate in the extraction process improved the yield of essential oil and the number of compounds in the extracts.

## Figures and Tables

**Table 1:** Extraction conditions used for each experiment.

Method	Sample Code	Temperature	Time (h)	Solvent	Solvent: Nutmeg Ratio	Magnesium Aluminometasilicate: Nutmeg Ratio
**Maceration**	M1	ambient	72	purified water	20:1	-
**Ultrasound-Assisted Extraction**	UAE1	25 °C	0.5	ethanol 50%	20:1	-
	UAE2			ethanol 70%	20:1	-
	UAE3			ethanol 96%	20:1	-
	UAE4			ethanol 70% + 0.5% magnesium aluminometasilicate	20:1	10:1
	UAE5			ethanol 70% + 1% magnesium aluminometasilicate	20:1	5:1
	UAE6			ethanol 70% + 2% magnesium aluminometasilicate	20:1	2.5:1
**Hydrodistillation**	HD1	100 °C	4	purified water	20:1	-
	HD2			purified water + 0.5% magnesium aluminometasilicate	20:1	10:1
	HD3			purified water + 1% magnesium aluminometasilicate	20:1	5:1
	HD4			purified water + 2% magnesium aluminometasilicate	20:1	2.5:1

**Table 2:** Chemical composition of nutmeg extracts and essential oils.

Compound	Sample Code ^a^											
	Essential Oil Compounds					Extracts Compounds						
	RI ^d^	HD ^e^ 1 (%)	HD2 (%)	HD3 (%)	HD4 (%)	M ^f^ 1 (%)	UAE ^g^ 1 (%)	UAE2 (%)	UAE3 (%)	UAE4 (%)	UAE5 (%)	UAE6 (%)
4-Carene, trans -(+)-	911	7.77	-	-	-	3.37	1.19	6.56	13.22	-	-	-
α-Thujene	928	0.99	1.04	0.93	0.99	-	2.63	0.72	1.42	0.84	0.92	0.81
α-Pinene	934	8.27	10.04	15.05	11.5	-	2.4	8.3	14.67	9.65	8.13	9.94
Camphene	944	-	2.8	0.11	0.11	-	-	-	-	-	-	0.15
Orthodene ^b^	949	0.51	-	-	-	-	-	-	0.59	-	-	-
3-Carene	956	0.53	-	-	-	-	-	0.42	0.78	-	-	-
α-Phellandrene	957	-	-	-	-	13.04	-	1.41	2.38	-	-	-
β-Myrcene	958	-	-	-	-	4.6	-	-	-	9.47	7.26	9.19
2-Carene	960	1.99	-	-	-	-	-	0.75	1.15	-	-	-
Sabinene	969	6.53	49.64	61.42	47.1	-	-	-	-	29.9	31.74	32.69
β-Pinene	970	26.61	4.03	4.32	3.84	-	-	-	-	-	-	-
Myristicin	981	3.26	2.34	2.15	2.16	-	-	-	-	1.79	1.65	1.96
γ-Terpinene	993	-	0.78	0.68	0.73	-	-	-	-	0.68	1.65	0.71
α-Terpinene	999	1.23	1.23	0.94	1.23	-	-	0.6	1.07	0.67	1.18	0.69
3,7,7-trimethylcyclohepta-1,3,5-triene	1005	0.18	0.47	0.33	0.44	-	-	-	-	0.37	-	-
Limonene	1009	-	5.62	4.2	5.23	-	-	-	-	4.2	4.46	4.63
Isoterpinolene	1030	-	2.11	1.18	2.07	-	-	-	-	1.19	2.02	1.22
*Cis*-sabinen hydrate	1037	7.76	0.58	0.3	0.83	-	-	-	-	1.3	2.32	1.46
γ-Terpineol	1043	-	0.04	0.04	0.04	-	-	-	0.14	-	-	0.09
α-Terpinolene	1051	-	0.7	0.38	0.7	-	-	-	-	-	-	0.54
Sylvestrene	1059	-	-	-	-	1.57	-	-	-	-	-	-
Isomethyleugenol	1062	0.2	-	-	-	6.38	-	-	-	-	-	-
*Cis*-p-menth-2-en-1-ol	1076	2.02	0.38	0.37	0.38	0.43	3.27	5.88	3.25	1.32	2.42	1.62
4-Propenyl syringol	1083	-	-	-	-	-	2.16	1.55	-	-	-	-
Citronellyl Decanoate ^b^	1110	0.3	-	-	-	-	-	0.43	0.16	-	-	-
1,1-dimethyl-2-[(1E)-3-methylbuta-1,3-dienyl cyclopropane	1116	-	-	-	-	-	8.4	29.99	47.32	-	-	-
Bicyclogermacrene	1125	0.29	-	-	-	-	-	-	-	-	-	-
4-Terpineol	1152	0.74	0.34	0.03	0.35	-	-	0.77	0.3	0.47	0.47	0.47
Cubebol ^b^	1174	0.05	-	-	-	-	-	-	-	-	-	-
Cubenene ^b^	1177	0.07	-	-	-	-	-	-	-	-	-	-
Piperitol	1189	0.09	-	-	-	-	-	-	-	-	-	-
Isoelemicin	1217	5.98	-	-	-	-	62.24	23.-63	-	21.5	21.5	18.85
Copaene	1228	-	0.25	0.01	0.25	-	-	-	-	-	-	-
β-Copaene ^b^	1233	0.25	-	-	-	-	-	-	-	-	-	-
γ-Amorphene ^b^	1245	-	0.75	0.03	0.78	-	-	-	-	0.43	0.92	0.08
*Cis*-α-bergamotene ^b^	1283	-	0.09	0.08	0.1	-	-	-	-	-	-	-
Isogermacrene	1311	-	0.99	0.01	1.15	1.61	-	-	-	0.86	2.44	0.82
Licarin B	1328	-	-	-	-	-	-	0.72	-	0.71	-	-
Bergamotene ^b^	1339	0.07	-	-	-	-	-	-	-	-	-	-
γ-Asarone	1361	3.69	1.21	1.22	3.51	0.79	0.84	2.07	-	0.82	1.84	1.25
Elemicin	1542	-	-	-	-	13.99	-	-	-	-	-	-
Myrislignan	2946	-	-	-	-	22.59	-	-	-	-	-	-
**Total Identified Compounds % ^c^**		79.38	85.43	93.78	83.6	68.37	83.13	83.8	86.45	86.1	90.92	87.17

All data are given with an 0.05 accuracy. ^a^ Sample code presented in Table 1. ^b^ First time chemical compounds extracted from nutmeg. ^c^ Quantity of all of the identified compounds. ^d^ Retention indices using RTX-5MS column and n-alkanes (C_9_–C_22_) as references. ^e^ HD–hydrodistillation. ^f^ M–maceration. ^g^ UAE–ultrasound-assisted extraction.
